# Greater Occipital Nerve Block as Preventive Treatment in Migraine: A Time‐To‐Event Analysis

**DOI:** 10.1111/papr.70041

**Published:** 2025-05-16

**Authors:** Giada Giuliani, Alessandro Viganò, Federica Papini, Barbara Petolicchio, Massimiliano Toscano, Edoardo Vicenzini, Vittorio Di Piero, Marta Altieri

**Affiliations:** ^1^ Department of Human Neuroscience Sapienza University of Rome Rome Italy; ^2^ IRCCS – Fondazione Don Carlo Gnocchi Milan Italy; ^3^ Department of Neurology University of Piemonte Orientale Novara Italy; ^4^ Department of Neurology and Neurophysiopathology Sandro Pertini Hospital Rome Italy; ^5^ Department of Neurology Fatebenefratelli Hospital‐Gemelli Isola Rome Italy; ^6^ University Consortium for Adaptive Disorders and Head Pain (UCADH) Pavia Italy

**Keywords:** combination therapy, difficult‐to‐treat migraine, GON block, MOH, response predictors

## Abstract

**Introduction:**

Greater occipital nerve block (GON‐B) may still represent a valuable strategy in migraine prophylaxis despite the development of newer drugs. The absence of a standardized method leads to variable outcomes and limits its use. In this light, we investigated GON‐B effects in a migraine population trying to define its duration and response predictors.

**Methods:**

In this real‐world study, we recruited patients with migraine who underwent bilateral GON‐B. They were clinically evaluated at baseline and then monthly for the next 3 months, using a 30‐day headache diary. The potential role of baseline headache characteristics in predicting treatment response was thoroughly analyzed.

**Results:**

A total of 73 patients were enrolled: 50 (68%) were affected by chronic migraine while 38 (52%) by medication overuse headache (MOH). The greatest clinical benefit due to GON‐B was reported during the first month, although the therapeutic response was substantially maintained during follow‐up. Notably, similar outcomes were recorded in patients with and without MOH. The mean duration of effect was 55.3 ± 72.0 days, with no clinical variables significantly influencing this parameter. The therapeutic response seemed to be stronger in patients with migraine with aura.

**Conclusions:**

GON‐B appears an effective option in migraine prophylaxis, even in difficult‐to‐treat patients. Its rapid effect, high tolerability, and cost‐effectiveness represent indisputable advantages. A prolonged duration of action, which could be favored by the combination of anesthetic and steroid and needs to be confirmed in future studies, may further optimize patient management.

**Abbreviations**
CGRP: calcitonin gene‐related peptide
CM: chronic migraine
CRs: complete responders
EM: episodic migraine
GON: greater occipital nerve
GON‐B: greater occipital nerve block
MAMs: monthly acute medications
MHDs: monthly headache days
MOH: medication overuse headache
NRs: non‐responders
PRs: partial responders
RCTs: randomized clinical trials
SD: standard deviation

## 1 Introduction

Migraine affects approximately 15% of the global population and is the leading cause of disability in people younger than 50 years old [1]. It typically begins during adolescence or early adulthood and persists throughout much of a patient's life, with a significant burden on healthcare systems [2].

Based on attack frequency, migraine is classified into chronic migraine (CM) (≥ 15 headache days per month, with at least eight having migraine characteristics) or episodic migraine (EM) (≤ 14 headache days per month), though the latter lacks a precise definition [3, 4]. CM affects about 2% of the general population and is responsible for frequent neurological and emergency department visits due to its difficult management. The development of medication overuse headache (MOH) can further complicate this condition [5].

Over the years, different prophylactic strategies have emerged to reduce migraine‐related disability. New migraine‐specific drugs targeting the calcitonin gene‐related peptide (CGRP) pathway have revolutionized disease management, going beyond the limits of traditional preventive treatments originally designed for other conditions [6, 7]. The monoclonal antibodies anti‐CGRP(−R) have proven to be a valid and safe option, with high effectiveness even in patients with prior treatment failures or concurrent MOH. Despite this, up to one‐third of patients with migraine could not respond optimally to these novel therapies [8]. Central sensitization, neuropeptides other than CGRP, and the presence of comorbidities might contribute to suboptimal responses. Additionally, fluctuations in treatment efficacy, influenced by endogenous and exogenous factors, can cause unpredictable worsening with feelings of distrust and frustration.

Greater occipital nerve block (GON‐B) is a peripheral nerve block technique, performed with local anesthetics alone or with a steroid‐local anesthetics combination. The greater occipital nerve (GON), the main sensory nerve of the occipital area, consists primarily of fibers from the second cervical dorsal root: its afferents, along with trigeminal nerve fibers, synapse with second‐order neurons in the spinal trigeminal nucleus and its cervical extension, which constitute the trigeminocervical complex. By modulating impulses within the trigeminal system and influencing central pain transmission, GON‐B offers a rationale for its use in migraine prophylaxis [9, 10].

This procedure, with documented effectiveness [11], has slowly reached a place in the preventive migraine armamentarium. GON‐B significantly reduces the frequency, duration, and intensity of attacks [12, 13]; it also mitigates migraine‐related disability, acting on the associated symptoms and decreasing analgesic intake [14, 15]. Its safety, cheapness, and the low risk of drug interactions represent additional advantages.

Despite the positive results from randomized clinical trials (RCTs), GON‐B remains a poorly standardized procedure. Controversies concern the correct methodology (such as the choice of drugs) and the duration of the effect, which determines the repetition frequency of administrations. While open‐label and retrospective studies have shown its efficacy in both EM and CM, little is known about clinical factors influencing response, such as baseline monthly headache days (MHDs), a factor capable of predicting the outcome in other injective therapies [16].

To address these gaps, we conducted a real‐world pragmatic study to evaluate the duration of the effect and possible response predictors of GON‐B in a migraine population. Our sample mainly consisted of difficult‐to‐treat patients, who are more likely to refer to specialized centers and receive injective treatment.

## 2 Methods

This single‐center, real‐life study was conducted at a tertiary headache center. The local Ethics Committee approved the study in the context of a larger study on predictive factors for migraine preventive treatments, and all patients provided written informed consent to participate. The study was conducted in accordance with the Helsinki Declaration.

### 2.1 Subjects and Experimental Design

We recruited migraine patients over 18 years of age who underwent GON‐B as preventive therapy. Treated patients were affected by EM or CM, with or without aura, according to the criteria of the International Classification of Headache Disorders. Patients with MOH were also included [3]. Exclusion criteria were (1) presence of different primary headaches (e.g., trigeminal autonomic cephalalgias); (2) other neurological and psychiatric conditions, such as epilepsy; (3) hypersensitivity to lidocaine or betamethasone; (4) medical contraindications to GON‐B (local infection on the injection site, open skull defect, skull fractures or skull abnormalities, subjects with any bleeding diathesis, history of brain traumatic injury). Being a pragmatic study, the concomitant use of ongoing preventive therapy was not considered an exclusion criterion because a complete wash‐out of undergoing migraine prophylaxis was not always possible. However, prophylactic treatment had to be stable for at least 3 months, with no changes in dosage either before or after the GON‐B. At the time of the study, most participants did not meet the reimbursement criteria for monoclonal antibodies anti‐CGRP(−R) in our country and were therefore receiving standard prophylactic medications.

According to our clinic's protocol, bilateral GON‐B was performed in patients with positive GON tenderness. It was repeated every 3 months when effective, while a watch‐and‐wait approach was preferred in patients who achieved a sustained complete response. Patients who continued to benefit from GON‐B at 3 months were clinically followed up to 6 months and then censored.

The injection, made with a 21‐gauge needle, consisted of 4 mg of betamethasone sodium phosphate (2 mL) and 2% xylocaine (1 mL) into the perineural space, midway between the inion and the mastoid.

At baseline (T0), the day of GON injection, the number of headache days in the previous month was recorded. Then, patients were evaluated monthly for the next 3 months (T1, T2, T3). During the study period, they had to fill in a 30‐day headache diary recording the number and duration of attacks, headache intensity, associated symptoms, and the intake of acute medications. Only patients with complete clinical reports were analyzed.

### 2.2 Headache Evaluation

A positive family history of headache, age at migraine onset and migraine duration (in years) were recorded. Pain characteristics including side, lateralization, quality, and severity were collected, indicating the presence of a more affected side. Patients had to rate their pain intensity (PI) using the Numeric Rating Scale, pointing out a value on a scale from 0 (“no pain”) to 10 (“worst possible pain”) [17]. The duration and frequency of attacks, along with associated symptoms, were documented. The presence of aura was noted, and patients were classified as episodic or chronic based on their MHDs. Additionally, we recorded the use of acute medications (MAMs), with the monthly number of pills taken, highlighting the presence of MOH. Current and previous prophylactic therapies were detected. All patients underwent a neurological exam; GON tenderness was considered an essential element for treatment, noting the presence of a more affected side, if any.

### 2.3 Outcome Variables and Statistical Analysis

Category data were presented as absolute or relative numbers or percentages. Continuous data were presented as mean ± standard deviation (SD), or median and first and third quartiles (Q1–Q3). Three main outcomes for the analysis were considered (MHDs, PI, MAMs). Baseline characteristics were used as predictors of the GON‐B effect, and responders' classes (complete responder [CR], partial responder [PR], and non‐responder [NR]) were used for response sub‐analysis. CRs, PRs and NRs were defined according to International Headache Society criteria [18]. One‐way ANOVA was used to test differences among baseline variables or responders' classes. The time effect of GON‐B was investigated through a mixed linear regression model for repeated measures of the same subject. Time was considered as within effect and baseline (tenderness, MOH, or pain laterality) or responders (CRs, PRs, and NRs). The interaction variable time was added. The patient was considered as a random effect. Chi‐square or Fisher's exact test (including the Fisher–Freeman–Halton's extension when needed) was used for categorical variables according to the number of subjects per cell. The duration of the GON‐B effect was investigated with the Kaplan–Meier curve to test differences in crude cumulative survival of the entire group and among specific responder profiles after the first administration (CRs vs. PRs vs. NRs). Gehan's test was used to test a significant difference among curves. Cox's proportional hazards regression model was used to evaluate the hazard ratios, with respective two‐sided 95% confidence intervals, between clinical baseline factors and time of GON‐B efficacy. Stata 16.2 and STATISTICA 7.0 software were used for data analysis, with a *p*‐value < 0.05 considered statistically significant.

## 3 Results

### 3.1 Demographic and Headache Characteristics at Baseline

We included seventy‐three patients in the analysis; their clinical characteristics are summarized in Table 1. Sixty‐three patients (86%) were female; the mean age was 55 ± 12 years. The median duration of the disease was 33 (15–42) years. Fifty (68.5%) subjects were affected by CM while eighteen (25%) had a high‐frequency EM. Out of 73, 11 (15%) had a diagnosis of migraine with aura and 38 (52%) had MOH. The mean duration from the beginning of the MOH was 21.82 ± 43.69 months. About 44% of subjects report unilateral pain, of whom 63% experience left‐sided lateralization. The mean PI was 8.47 ± 1.24 while the mean number of MHDs was 18.68 ± 8.10. The presence of bilateral pain was associated with a higher frequency of attacks (*p* = 0.004). The mean number of MAMs was 19.38 ± 18.16. Forty‐two (57.5%) patients were undergoing concurrent prophylactic therapy while the median number of previous migraine prophylaxis was 2 (1–3). Bilateral GON tenderness was reported by 76% of patients and left‐sided by 14%.

**TABLE 1:** Headache characteristics at baseline (T0).

Age	(Mean, SD)	55.03	11.63
	(Median, Q1–Q3)	56	48–60
Migraine duration (years)	(Mean, SD)	31.34	15.76
	(Median, Q1–Q3)	33	15–42
CM	(*n*, %)	50	68.49
MHDs	(Mean, SD)	18.68	8.10
	(Median, Q1–Q3)	20	11–28
Pain lateralization			
Unilateral	(*n*, %)	32	43.84
Bilateral		35	47.95
Both		6	8.22
Unilateral pain			
Right	(*n*, %)	12	37.50
Left		20	62.50
PI (NRS)	(Mean, SD)	8.47	1.24
Accompanying symptoms			
Photophobia/phonophobia	(*n*, %)	50	68.5%
Nausea and/or vomiting		33	45.2%
MAMs	(Mean, SD)	19.38	18.16
	(Median, Q1–Q3)	12	8–25
Acute medications			
NSAIDs	(*n*, %)	53	72.6
Triptans		43	58.9
Previous preventives	(Mean, SD)	2.37	1.93
	(Median, Q1–Q3)	2	1–3
MOH	(*n*, %)	38	52.05

Abbreviations: NRS, Numeric Rating Scale; NSAIDs, nonsteroidal anti‐inflammatory drugs.

### 3.2 Efficacy

After GONB, about 46% of patients reported headache improvement. Thirty‐five patients repeated the treatment after 3 months: twenty‐one of them still experienced a benefit after the second administration (the effect of GON‐B repetition was not analyzed). Only three subjects reported adverse events, all of short duration. One of them described pain at the injection site after the first injection while two had respectively a feeling of unsteadiness and allodynia after the second administration.

On the entire group of patients, an overall significant reduction in MHDs was observed over time [*F*(3, 216) = 34.931; *p* = 0.00001] at monthly time points. At T1, we observed the best response to GON‐B compared to the baseline in terms of MHDs that reached the absolute minimum (11.37 vs. 18.68, *p* < 0.001). At T2 and T3 follow‐ups, the number of MHDs increased compared to T1, but it remained lower than the initial value (Table 2).

**TABLE 2:** Change in MHDs after GON‐B.

MHDs	Mean	SE	*p*
T0	18.68	1.02	
T1	11.37	1.02	< 0.001
T2	13.36	1.02	< 0.001
T3	13.78	1.02	< 0.001

The change in MHDs compared to the baseline was significant at every time point regardless of clinical characteristics, except for the GON tenderness on the right side that was associated with a lack of response at T3 (*p* = 0.132). Similarly to MHDs, PI also showed an overall significant decrease [*F*(3, 216) = 15.28, *p* = 0.000001] with the absolute minimum at T1 (T0: 8.47 vs. T1: 7.33; Table 3). As for MHDs, PI reduction was also significant in patients both with positive bilateral and left GON tenderness, but not in patients with positive right GON tenderness, with respect to baseline. The T3–T0 PI difference in the group with left GON tenderness was significantly greater compared to the group with bilateral tenderness (*p* = 0.001). The PI change at T3 compared to the baseline was not significant in patients with right‐sided headache (*p* = 0.083).

**TABLE 3:** Change in PI after GON‐B.

PI	Mean	SE	*p*
T0	8.47	0.18	
T1	7.33	0.18	< 0.001
T2	7.59	0.18	< 0.001
T3	7.58	0.18	< 0.001

The mean number of MAMs significantly decreased after treatment [*F*(3, 216) = 17.37, *p* = 0.000001], reaching its minimum level at T1 (11.12 vs. 19.38, *p* < 0.001). Despite slightly higher analgesic consumption, the benefit remained significant at subsequent time points (Table 4). Tenderness lateralization was not associated with the GON‐B effect on MAMs [*F*(9, 207) = 1.28; *p* = 0.24]. As well, in patients with right‐sided headache, the reduction in analgesic consumption was not significant at T2 (*p* = 0.062) and T3 (0.076) follow‐up compared to baseline. The presence of a higher consumption of analgesics in patients with MOH did not affect GON‐B effectiveness, with a significant reduction at each time point. Overall, the response to treatment did not differ in the patients with and without MOH.

**TABLE 4:** Change in MAMs after GON‐B.

MAMs	Mean	SE	*p*
T0	19.38	1.59	
T1	11.12	1.59	< 0.001
T2	12.25	1.59	< 0.001
T3	12.85	1.59	< 0.001

### 3.3 Time‐To‐Event Analysis: Duration of the Effect and Factors Influencing the Time Length of the GON‐B

The mean duration of the GON‐B in the entire sample was 55.3 ± 72.0 days. No difference was found in curves between patients with versus without prophylaxis (*Z* = 0.34, *p* = 0.72), CM versus EM (*Z* = 1.37, *p* = 0.16) or patients with or without MOH (*Z* = 1.37, *p* = 0.16), nor in tenderness location (*χ* ^2^ = 3.78, *p* = 0.28). The pain localization did not affect the duration of the response to GON‐B (*χ* ^2^ = 3.51, *p* = 0.48). The presence of aura only provided a trend to significance (*Z* = 1.65, *p* = 0.09).

**FIGURE 1:**
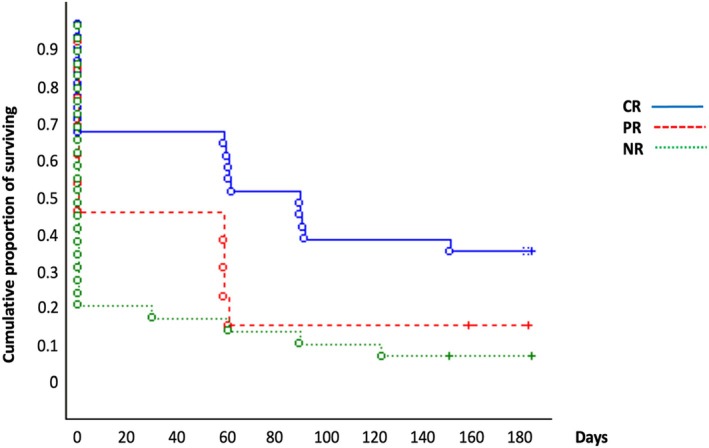
Kaplan–Meier curve according to the first response category.

On the other hand, the magnitude of the response in the first month after the GON‐B (namely CR vs. PR vs. NR) provided a significant difference in the trajectory of the duration of GON‐B (*χ* ^2^ = 14.67, *p* = 0.0002). In particular, CR had a significantly different trajectory compared with PR and NR, who did not differ from each other (Figure 1).

### 3.4 Predictors of Improvement

In the univariate analysis, the presence of aura was associated with a better response to GON‐B and a longer effect of the clinical benefit in terms of reduction of MHDs [*F*(3, 213) = 3.52, *p* = 0.02]. However, the difference between groups was significant only at the last time point (14.63 ± 9.59 vs. 8.4 ± 5.23 *p* = 0.049). By contrast, photophobia and phonophobia were not associated with a larger MHDs reduction [*F*(3, 213) = 1.82, *p* = 0.14]. As well, nausea was not associated with a different response [*F*(6, 210) = 1.226, *p* = 0.29] and also the absence of MOH was not associated with a better response [*F*(3, 213) = 0.385, *p* = 0.76]. Regarding preventive and acute treatments, neither the presence of other preventive therapy [*F*(3, 186) = 1.04, *p* = 0.41] nor the type of acute medication used [*F*(15, 201) = 1.087, *p* = 0.29] impacted the reduction of MHDs.

Regarding changes in PI and MAMs, none of the clinical characteristics had any association with a reduction in the outcome.

At the multivariate analysis by Cox's proportional hazards regression, the model showed that none among the migraine subtypes (CM vs. EM), aura, years since migraine onset, pain localization, photophobia and phonophobia, nausea/vomiting, time since MOH and migraine chronification, current prophylaxis, and lateralization of GON tenderness had a clinical impact on the temporal duration of the GON‐B effect (*χ* ^2^ = 7.91, *p* = 0.791, Table 5) neither on the entire sample nor on the responders rate (*χ* ^2^ = 8.56, *p* = 0.74, Table 6).

**TABLE 5:** Predictors of GON‐B duration in the entire group.

Variables	Beta	*t*‐value	Wald	*p*
Time since migraine onset (years)	−0.001047	−0.10763	0.011584	0.914292
CM versus EM	0.023885	0.07010	0.004914	0.944112
Aura	−0.181370	−0.43999	0.193590	0.659949
Pain localization	−0.516783	−1.67740	2.813669	0.093474
Pain lateralization	0.000423	0.12800	0.016383	0.898153
Photo/phonophobia	−0.249706	−0.59511	0.354153	0.551776
Nausea/vomiting	−0.000153	−0.04847	0.002349	0.961344
MOH	0.616805	1.81738	3.302886	0.069168
Time since MOH onset (months)	−0.003537	−0.58669	0.344206	0.557415
Time since CM onset (month)	‐0.000319	‐0.05885	0.003463	0.953073
Current prophylaxis (Y/N)	0.013867	0.04502	0.002026	0.964095
Tenderness side (R/L/B)	‐0.100290	‐0.74712	0.558182	0.454999

**TABLE 6:** Predictors of GON‐B duration according to the first time response.

Variables	Beta	*t*‐Value	Wald	*p*
Time since migraine onset (years)	−0.002394	−0.24972	0.062361	0.802805
CM versus EM	−0.268926	−0.73852	0.545407	0.460206
Aura	−0.133883	−0.31714	0.100576	0.751142
Pain localization	−0.476593	−1.53176	2.346273	0.125593
Pain lateralization	0.000563	0.16820	0.028292	0.866426
Photo/phonophobia	−0.487593	−1.10220	1.214851	0.270382
Nausea/vomiting	0.000130	0.03993	0.001594	0.968152
MOH	0.584789	1.72100	2.961828	0.085261
Time since MOH onset (months)	−0.004243	−0.67478	0.455331	0.499819
Time since CM onset (month)	−0.001732	−0.31074	0.096556	0.756004
Current prophylaxis (Y/N)	−0.317024	−0.97506	0.950733	0.329540
Tenderness side (R/L/B)	−0.064994	−0.46666	0.217770	0.640747

## 4 Discussion

This article yielded evidence on the efficacy of GON block as preventive therapy for difficult‐to‐treat migraine forms. Our population was mainly affected by high‐frequency EM or CM: these patients generally experience high levels of headache‐related disability with a significant risk of refractory migraine [19]. More than half were on prophylactic therapy at the time of the GON‐B (four patients were taking two oral prophylactic drugs), with an average of two prior treatment failures. A combination therapy may be helpful in cases of suboptimal response, but it is often limited by issues of reimbursement and tolerability. Furthermore, lack of efficacy and adverse events play an essential role in poor treatment adherence [20]: contrary to expectations, also the underuse of both acute and preventive medications contributes to migraine progression [21].

Although GON‐B is a valid strategy in migraine management [9, 12], it remains underutilized. The reasons for its low diffusion are to be found mainly in the (minimally) invasive nature and the poor standardization of the procedure. There is a great variability in used drugs, to which is added the uncertainty about the GON‐B effect size and duration. Our study aimed to provide insights to help fill this gap.

First, our research confirmed the GON‐B effectiveness. Half of the patients had a clinical benefit after the first injection with a very low rate of side effects: the rapid‐acting properties, as already observed in the acute treatment of migraine [22, 23], represent an undisputed advantage of this procedure. Analyzing the temporal trajectory of response in our patients, we found that the greatest benefit related to GON‐B for all clinical parameters (MHDs, PI, and MAMs) was reported during the first month. We also showed a similar efficacy in patients with and without MOH, as already documented in clinical trials and open‐label studies [24, 25]. Overall, the sample reduced the number of MHDs by about 40%.

The presence of concomitant prophylaxis did not affect the patient's response trajectory, suggesting that GON‐B can serve as an effective stand‐alone therapy [26]. Moreover, its mode of administration, that minimizes drug interactions, coupled with the absence of significant economic limitations, makes it a valuable option, even as an add‐on, for optimizing patient management.

In our population, the effect of a single GON‐B seemed to last an average of 55 days, with a persistent benefit of up to 3 months (although with a magnitude decrement). This result was longer than what is expected by RCTs, often performed using only anesthetic. A study on children and adolescents who underwent GON‐B with lidocaine and methylprednisolone showed that a percentage of treated patients were free from attacks for at least 3 weeks [27]. The benefit extended up to 9 weeks in selected cases. We might wonder if the addition of steroidal drugs might modify effect duration, with a longer‐lasting efficacy. Anesthetics primarily act by reducing nociceptive impulses that travel from the trigeminocervical complex to the higher centers involved in pain processing [28]; they can also decrease peripherally the CGRP release from trigeminal afferents [29]. On the other hand, steroids favor the reduction of neural inflammation, but they also modulate neural plasticity by acting on their receptors, located both in the peripheral and central nervous systems [30].

Stratifying our population based on the response rate, the only factor influencing a longer duration of the GON‐B effect is a better initial response. We could speculate that the greater response observed in subjects with CRs might be secondary to a stronger plasticity in pain circuits. It is known that GON‐B can increase serotonin transmission from the brainstem to cerebral cortices, facilitating the remodeling of brain circuits [31, 32]. Besides providing insights on pathophysiological reasons, a better definition of the GON‐B duration could help to identify the correct administration regimen, maximizing the benefit.

Additionally, we carefully analyzed headache features that could influence GON‐B effectiveness: interestingly, patients affected by migraine with aura presented a more marked reduction of MHDs. According to a previous case series, GON‐B seems to be effective as a stand‐alone treatment of prolonged migraine auras [33]. Larger studies are needed to evaluate if migraine with aura per se is a positive predictive factor for treatment response.

In literature, GON tenderness seems to be a predictive marker of efficacy, while in clinical practice it is often used as an inclusion criterion for selecting patients for the procedure [34, 35]. Curiously, the presence of a more tender spot on the right side seems to negatively affect the treatment response in our population, since patients with a right GON tenderness showed a nonsignificant change in MHDs at T3 compared to the baseline. Also, the presence of right‐sided attacks seemed to be associated with a lower GON‐B efficacy in our study. Unilateral pain is considered a hallmark of migraine [2], and patients frequently describe one side as more severely affected, even if both sides are involved. Pain localization can be a significant factor in the response to both acute and preventive migraine medications, with a greater benefit in patients experiencing unilateral rather than bilateral pain [36, 37]. Recent insights have shown that right‐sided and left‐sided migraine might not be identical, with specific findings in psychiatric, cognitive, and autonomic domains and neuroimaging [38]. Despite the presence of a right or left phenotype might have clinical relevance affecting treatment response, we cannot draw firm conclusions due to the small number of recruited patients; in fact, patients with right‐sided pain are the less represented group in our population, and therefore the lack of significance could be attributed to insufficient statistical power.

Finally, some other points need to be addressed. First, we cannot rule out that part of the observed response in CRs may be due to the placebo effect. The application of a drug to the head in the hospital setting has been associated with a placebo response superior to oral and subcutaneous administration and not inferior to intravenous injection [39], resulting in a greater reduction in the number of MHDs. Nevertheless, this is not a negative factor but rather another tool to optimize patient management.

Furthermore, the lack of a standardized procedure implies significant methodological heterogeneity and different administration protocols. In this sense, we cannot be sure that our results could be immediately generalizable to other centers. For instance, we included only patients with positive GON tenderness, but this factor is not always considered when the patient is referred for treatment. Studies on patients with and without GON tenderness are necessary to investigate the specific role of the lateralization of GON tenderness as a response predictor, excluding a potential ceiling effect. Similarly, the low number of clinical characteristics in our study that can influence GON‐B response makes it difficult to identify the ideal candidate for this treatment, but this element needs to be further analyzed in the future.

In conclusion, our real‐life study confirmed that GON‐B is an effective strategy in migraine treatment even in patients affected by debilitating forms of the disease. The efficacy is not influenced by the presence of MOH and other prophylactic therapies. Thanks to the rapid action, low cost, and minimal drug interactions, GON‐B might be a useful strategy in cases of unpredictable disease worsening or during periods of prophylaxis suspension with anti‐CGRP (mandated by regulatory authorities). We observed a mean duration of the GON‐B effect of 55 days, which appeared to be even longer in patients who experienced a more significant reduction in MHDs. Further studies are needed to improve the treatment protocol (e.g., administering GON‐B every 2 months) and refine patients' selection criteria (e.g., aura presence or response during the first month).

## Author Contributions

G.G.: conceptualization, data curation, investigation, writing – original draft preparation; A.V.: formal analysis, writing – original draft preparation; FP: data curation, writing – review and editing; B.P., M.T., E.V.: investigation; V.D.P.: validation, visualization, writing – review and editing; M.A.: supervision, writing – review and editing. All the authors have read and approved the final manuscript.

## Ethics Statement

This study complied with the Declaration of Helsinki; the ethics committee of Sapienza University of Rome, Italy, reviewed and approved the research protocol (Ref 4467). Written informed consent for participation was obtained from the subjects.

## Conflicts of Interest

The authors declare no conflicts of interest.

## Data Availability

The data that support the findings of this study are available on request from the corresponding author. The data are not publicly available due to privacy or ethical restrictions.
